# Editorial: Involvement of Blood Brain Barrier Efficacy, Neurovascular Coupling, and Angiogenesis in the Healthy and Diseased Brain, Volume II

**DOI:** 10.3389/fphys.2022.829901

**Published:** 2022-01-31

**Authors:** Daniela Carnevale, Fabrice Dabertrand, Clotilde Lecrux, Jean-Luc Morel

**Affiliations:** ^1^Department of Molecular Medicine, Sapienza University of Rome, Rome, Italy; ^2^Research Unit of Neuro and Cardiovascular Pathophysiology, IRCCS Neuromed, Pozzilli, Italy; ^3^Department of Anesthesiology, University of Colorado Anschutz Medical Campus, Aurora, CO, United States; ^4^Department of Pharmacology, University of Colorado Anschutz Medical Campus, Aurora, CO, United States; ^5^Montreal Neurological Institute, McGill University, Montreal, QC, Canada; ^6^Univ. Bordeaux, CNRS, IMN, UMR 5293, Bordeaux, France

**Keywords:** blood brain barrier, neurovascular coupling, neurodegenerative disorders, stroke, pericyte, endothelial cell, astrocyte, subarachnoid hemorrhage

The second volume of the special issues dedicated to the involvement of blood-brain barrier (BBB), neurovascular coupling, and angiogenesis in the regulation of brain functions contains four original articles, two reviews, and one perspective to complete the overview of the complexity of the neurovascular unit and its functions. The articles are more focused on astrocytes' participation in neurovascular coupling and on alteration after subarachnoid hemorrhage or in murine Alzheimer's disease models.

## Original Articles

In this issue, the control of the neurovascular unit by astrocytes is discussed in two articles. Firstly, the astrocyte expression of CDK5, known to participate in protecting the neurovascular unit during glutamate-induced excitotoxicity, regulates functional interaction between astrocytes and neurons as shown by the transplantation of CDK5 knock-out astrocytes in the hippocampus (Toro-Fernández et al.). This transplantation, inducing remodeling of astrocyte shape and modification of calcium signaling, affects the expression of synaptic proteins such as PSD95 or synapsin, suggesting the modification of neuronal activity in the hippocampus. These results should be taken into consideration for further investigation as a potential tool for cell transplantation to restore brain function after brain injuries.

The second study (Kim et al.) is focused on component 8 gamma (C8γ), known to inhibit neuroinflammation. The study specifies that C8γ is expressed by astrocytes of the neurovascular unit and antagonizes the sphingosine-1-phosphate receptor 2 (S1PR2) expressed on endothelial cells. Knockdown of C8γ enhances BBB permeability, whereas recombinant C8γ reduces neuroinflammation-induced BBB damage, thus confirming the crucial role of interactions between astrocytes and endothelial cells in neuroinflammation pathophysiology.

The hypothesis indicating that neurovascular coupling is affected in Alzheimer's disease is now well established in several animal models. However, the mechanisms altered in the different mouse models remain to be elucidated. In the APP mouse model (Li et al.), the increase of cerebral blood flow due to Shaffer collaterals stimulation is drastically reduced compared to those observed in wild type, and the properties of neuronal activity are altered in hippocampus slices. At the same time, gliovascular coupling is impaired as shown though alteration of vascular reactivity and both calcium and reactive oxygen species' signals in perivascular astrocytes as observed in hippocampus slices by two-photon microscopy.

Finally, subarachnoid hemorrhage (SAH) represents a severe form of stroke that induces neurological deficits long after the ictus and considerably alters the life of the patient. Koide and collaborators, through modeling of SAH in mice, investigate how SAH impacts cerebral autoregulation required to maintain brain perfusion. They have developed elegant new technics to calibrate *in vivo* laser Doppler flowmetry to access cerebral blood flow values (Koide et al.). This method allows for evaluation and validation of preclinical approaches to restore cerebral vascular autoregulation, which is impaired by several cardiovascular or cerebrovascular pathologies.

## Reviews and Perspectives

The review entitled “Pericytes: Intrinsic Transportation Engineers of the CNS Microcirculation” (Eltanahy et al.) perfectly completes the one published in the first volume of this special issue regarding pericytes (Hariharan et al., 2020). It summarizes both the functional particularities of brain pericytes in terms of calcium signaling and in terms of sensitivity to reactive oxygen species (ROS) differentiating them to smooth muscle cells of brain-penetrating arteries. Moreover, the authors propose to define the “Pericyte sensome” as “the range of ligands sensed by pericytes” and give pericytes a central role in neurovascular unit via their interactions with endothelial and immune cells to interpret the alteration of the capillary environment. Pericytes' role in the angiogenesis, sprouting, and stabilization of BBB has been evoked through their close relationships with endothelial cells, particularly via their ability to synthesize angiopoietin-1. The authors also propose to investigate some new research pathways to give new roles to pericytes in memory processes during sleep via their environmental sensor properties, and in pathological conditions like growth of glioblastoma or in BBB alteration after COVID-19 infection. They devote a chapter to “vascular resilience,” which is now proposed as a possible explanation for the progression of Alzheimer's disease. The experimental and conceptual elements that support this proposal are very clearly grouped and illustrated to show how and when alterations in pericyte functions could appear and participate in the pathology. This is followed by a chapter on the role of pericytes in stroke including the pericytes' specific reaction to oxygenation. The last part is focused on the putative targeting of pericytes in regenerative medicine and therapeutics for neurodegenerative disorders before concluding on the recent research in 3D culturing of organoids modeling neurovascular unit. As also evoked in the perspectives article showing the importance of the development of 3D systems in the modeling of the BBB (Galpayage Dona et al.), the authors remind us of the specificity of the endothelial cells of the cerebral microvessels, their polarity through their specific equipment in transporters and proteins of junctions, and their specific interactions with the pericytes and astrocytes to form the neurovascular unit. They analyze the advantages and disadvantages of the three main techniques of three-dimensional reproduction of intracerebral vascular networks in context of the challenge of stable perfusion of the network, the implementation of cellular interactions while preserving the vascular complexity, and allowing the expression of neuronal activity. They also insist on the importance of the shear stress and force applied on the 3D reconstructed vascular network and on the choice of the cell lineage to reproduce the diversity of situations observed in brain vasculature.

The last review (Bernier et al.) evokes the “geographic heterogeneity” of neurovascular units in the brain. Since the cell composition along the vascular tree is heterogenous, they argue that regional specificity of neurovascular unit compounds should be considered to propose new research pathways and be able to develop more acute therapeutic targets. In fact, the specific alteration of the white matter in small cerebrovascular diseases suggest vascular specificities of white matter as detailed in sections concerning each cell type of the neurovascular unit. They also point out differences between endothelial cells of the cortex and subcortical areas such as the hippocampus, the pericyte density in cortex layers, and differences in shapes and interactions between astrocyte and endothelial cells. These differences could explain the vulnerability of vascular function in specific regions. The last part of the review compares the *in vivo* methods to appreciate the dynamics of neurovascular unit function (multi-photon microscopy, miniaturized fluorescence microscopes, multi-fiber photometry and functional ultrasound imaging).

This second volume closes our Research Topic entitled *Involvement of Blood Brain Barrier Efficacy, Neurovascular Coupling, and Angiogenesis in the Healthy and Diseased Brain*. These articles and reviews have further highlighted the role of the brain microcirculation in important fundamental mechanisms in both health and disease. However, several gaps remain in our understanding. For example, the regional heterogeneity in the vascular function suggested by Li et al. and reviewed by Bernier et al. will require further investigations. New methods for *in vivo* investigation of deeper brain structure, like three-photon imaging, will help fill these gaps. However, technological advances for recording macro-scale signaling and mechanics, sites for angiogenesis, and new transgenic and conditional animal models are needed. Genetically encoded voltage sensors would also be a valuable tool to further advance the field. Finally, a better integration of biological variables, especially sex differences, would help to improve the translation from bench to bedside.

## Author Contributions

All authors listed have made a substantial, direct, and intellectual contribution to the work and approved it for publication.

## Funding

FD received awards from the CADASIL Together We Have Hope non-profit organization; a research grant from the Center for Women's Health Research located at the University of Colorado Anschutz Medical Campus; a research grant from the University of Pennsylvania Orphan Disease Center in partnership with the cureCADASIL, and the National Heart, Lung, and Blood Institute R01HL136636. J-LM received grants from Center National de la Recherche Scientifique (CNRS) and Centre National des études spatiales (CNES). DC received grants from the Italian Ministry of Health (MOH) for the Ricerca Corrente program.

## Conflict of Interest

The authors declare that the research was conducted in the absence of any commercial or financial relationships that could be construed as a potential conflict of interest.

## Publisher's Note

All claims expressed in this article are solely those of the authors and do not necessarily represent those of their affiliated organizations, or those of the publisher, the editors and the reviewers. Any product that may be evaluated in this article, or claim that may be made by its manufacturer, is not guaranteed or endorsed by the publisher.
